# Supplementary material for: Phenylalanine Ammonia Lyase GmPAL1.1 Promotes Seed Vigor under High-Temperature and -Humidity Stress and Enhances Seed Germination under Salt and Drought Stress in Transgenic Arabidopsis
Source: Plants (Basel). 2022 Nov 25;11(23):3239. doi: 10.3390/plants11233239 (PMC9736545; doi:10.3390/plants11233239)
Supplement: Supplementary file 1 [file plants-11-03239-s001.zip › Supplementary Figure S1.pdf]

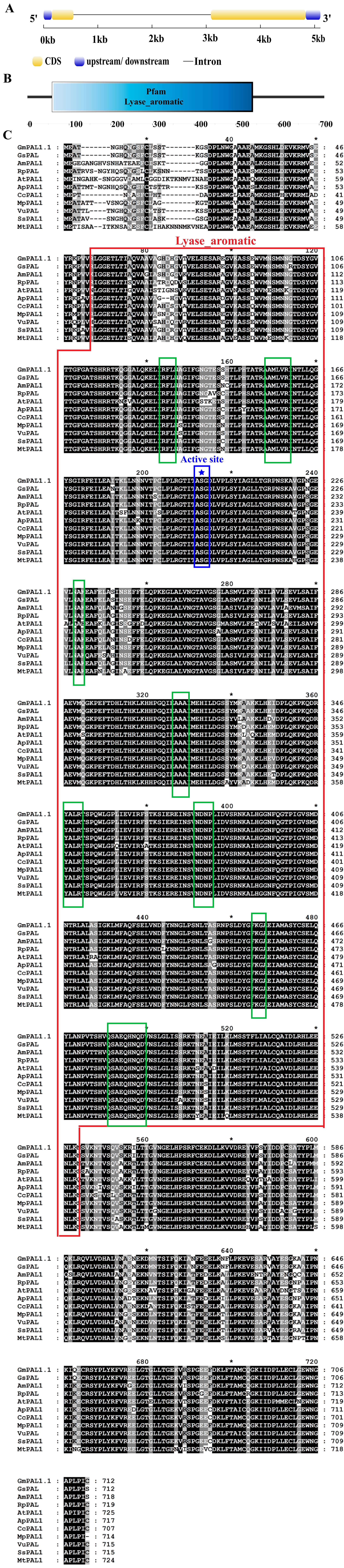

Supplementary Figure S1. Sequence analyses of the *GmPAL1.1* gene from the soybean cultivar Xiangdou No. 3. (A) Gene structure of *GmPAL1.1*. (B, C) Structure analysis of the GmPAL1.1 protein. The SSG triad motif and other conserved active sites were highlighted in blue and green. Gm, *Glycine max*; Gs, *Glycine soja*; Am, *Astragalus mongholicus*; Mt, *Medicago truncatula*; Rp, *Robinia pseudoacacia*; Ap, *Abrus precatorius*; Vu, *Vigna unguiculate*; Mp, *Mucuna pruriens*; Ss, *Spatholobus suberectus*; Cc, *Cajanus cajan*.
