# Supplementary material for: Phenylalanine Ammonia Lyase GmPAL1.1 Promotes Seed Vigor under High-Temperature and -Humidity Stress and Enhances Seed Germination under Salt and Drought Stress in Transgenic Arabidopsis
Source: Plants (Basel). 2022 Nov 25;11(23):3239. doi: 10.3390/plants11233239 (PMC9736545; doi:10.3390/plants11233239)
Supplement: Supplementary file 1 [file plants-11-03239-s001.zip › Supplementary Figure S2.pdf]

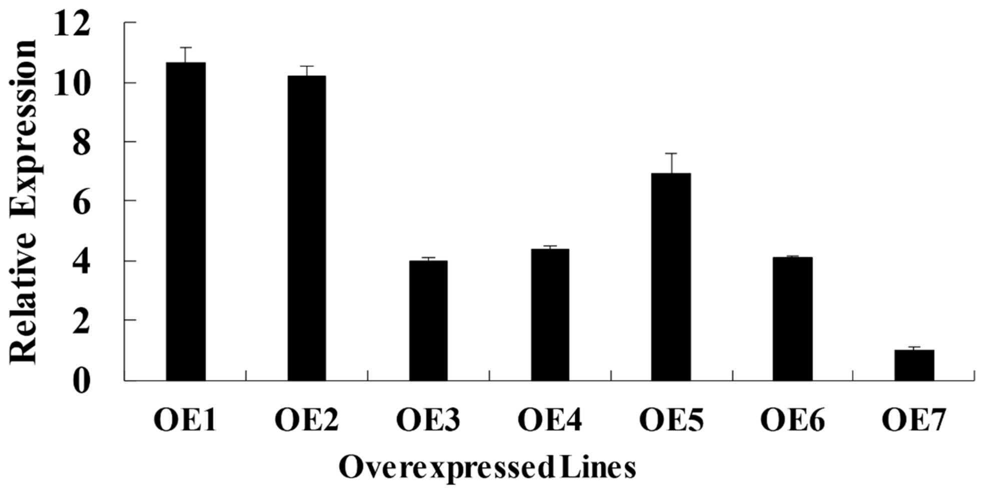

Supplemeantary Figure S2. Relative expression levels in leaves of *GmPAL1.1* overexpressing *Arabidopsis* lines. The surface-sterilized *Arabidopsis* seeds of the wild type and transgenic lines were planted on 1/2 MS medium for 7 d. The 7-day-old transgenic and wild-type seedlings were transferred to pots for another 3 weeks growth. RNA was extracted from the leaves of positive *Arabidopsis thaliana* and used for qRT-PCR analysis. The sample with the lowest expression of *GmPAL1.1* was used as the control, and $2^{-\Delta\Delta CT}$ was used for quantitative analysis method. Data was expressed as the mean value ± SD of three independent biological repeats.
