# Supplementary material for: Phenylalanine Ammonia Lyase GmPAL1.1 Promotes Seed Vigor under High-Temperature and -Humidity Stress and Enhances Seed Germination under Salt and Drought Stress in Transgenic Arabidopsis
Source: Plants (Basel). 2022 Nov 25;11(23):3239. doi: 10.3390/plants11233239 (PMC9736545; doi:10.3390/plants11233239)
Supplement: Supplementary file 1 [file plants-11-03239-s001.zip › Supplementary Figure S3.pdf]

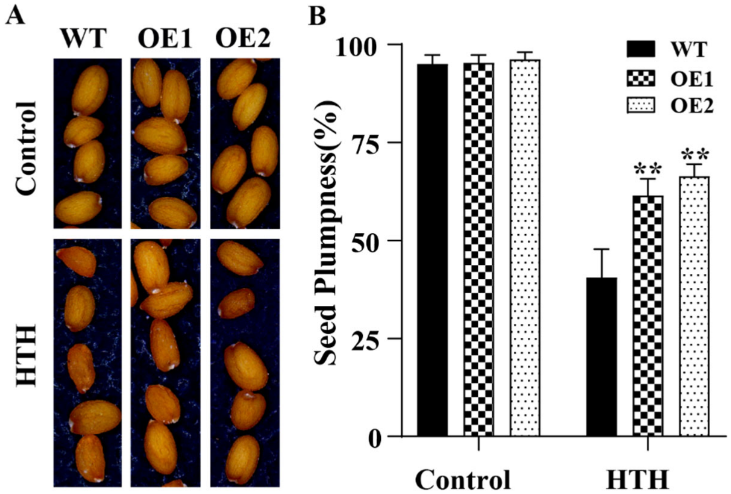

Supplementary Figure S3. Seed plumpness of WT and OE seeds after HTH stress. (A,B) the mature seeds harvested from the *GmPAL1.1* overexpressing *Arabidopsis* (OE) lines and WT after treated under normal condition and HTH stress at the physiological maturity stage, prespectively. Seeds were photographed using a MVX10 stereo fluorescence microscope (Olympus, Tokyo, Japan) and the number of plump seeds was counted.
