# Supplementary material for: Phenylalanine Ammonia Lyase GmPAL1.1 Promotes Seed Vigor under High-Temperature and -Humidity Stress and Enhances Seed Germination under Salt and Drought Stress in Transgenic Arabidopsis
Source: Plants (Basel). 2022 Nov 25;11(23):3239. doi: 10.3390/plants11233239 (PMC9736545; doi:10.3390/plants11233239)
Supplement: Supplementary file 1 [file plants-11-03239-s001.zip › Supplementary Figure S4.pdf]

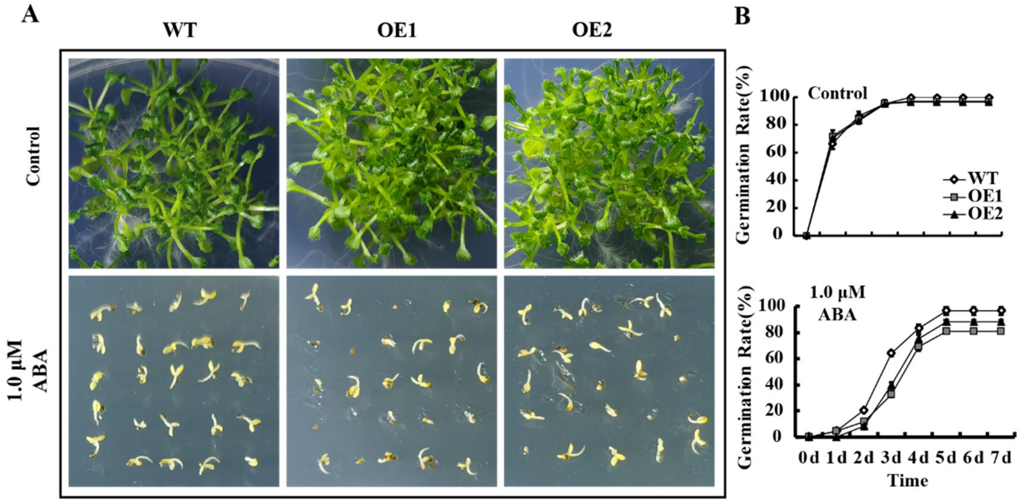

Supplementary Figure S4. Germination rate of the mature seeds of WT and OE lines under ABA treatment. Germination rate of the mature seeds harvested from the *GmPAL1.1* overexpressing *Arabidopsis* (OE) lines and WT grown under normal condition. (A) Germination performance of the WT and OE lines under ABA treatment. The seeds of the WT and OE lines were grown for 7 d under 1.0 μM ABA condition. (B) Germination rate of WT and OE lines under 1.0 μM ABA condition. The germination rates were recorded on the 7th d. Each experiment was repeated three times, with each replicate containing 50 seeds. Values are mean ± SD of three biological replicates. ** indicated significant differences at 1% probability level between WT and transgenic *Arabidopsis* lines.
