# Supplementary material for: Phenylalanine Ammonia Lyase GmPAL1.1 Promotes Seed Vigor under High-Temperature and -Humidity Stress and Enhances Seed Germination under Salt and Drought Stress in Transgenic Arabidopsis
Source: Plants (Basel). 2022 Nov 25;11(23):3239. doi: 10.3390/plants11233239 (PMC9736545; doi:10.3390/plants11233239)
Supplement: Supplementary file 1 [file plants-11-03239-s001.zip › Supplementary Figure S5.pdf]

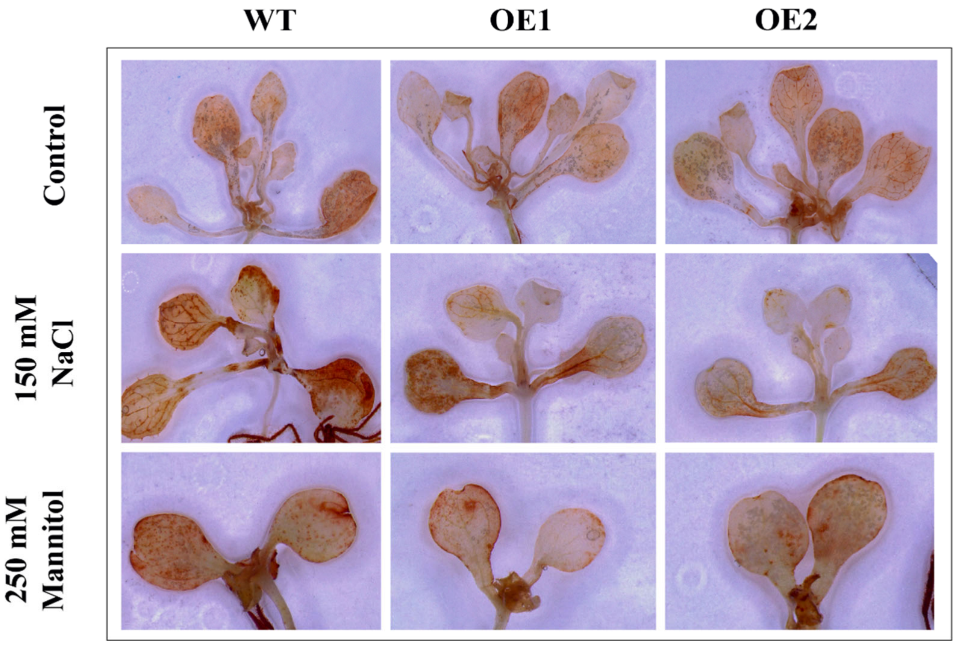


**Supplementary Figure S5**. DAB staining of 7-day-old *Arabidopsis* seedlings. Wild-type (WT) and transgenic (OE1, OE2) *Arabidopsis* seeds were exposed on 1/2 MS medium (control condition) or NaCl and Mannitol conditions for 7 d. The 7-day-old seedlings were used for DAB staining. Values are mean ± SD of three biological replicates.
